# Role of Liquid Biopsy in Progressive PSA Patients after Radical Prostatectomy

**DOI:** 10.3390/diagnostics14202293

**Published:** 2024-10-16

**Authors:** Marcel Figueras, Lourdes Mengual, Mercedes Ingelmo-Torres, Fiorella L. Roldán, Bernat Padullés, Héctor Alfambra, Sandra Herranz, Pilar Paredes, Gary Amseian, Joel Mases, Maria J. Ribal, Laura Izquierdo, Antonio Alcaraz

**Affiliations:** 1 Department and Laboratory of Urology, Hospital Clínic Barcelona, 08036 Barcelona, Spain; mfiguerast@recerca.clinic.cat (M.F.);; 2 Genetics and Urological Tumours, Institut d’Investigacions Biomèdiques August Pi i Sunyer (IDIBAPS), 08036 Barcelona, Spain; 3 Department of Biomedical Sciences, Faculty of Medicine and Health Sciences, Universitat de Barcelona (UB), 08036 Barcelona, Spain; 4 Department of Nuclear Medicine, Hospital Clínic Barcelona, 08036 Barcelona, Spain; 5 Department of Clinical Fundamentals, Faculty of Medicine and Health Sciences, Universitat de Barcelona (UB), 08036 Barcelona, Spain; 6 Department of Radiology, Hospital Clínic Barcelona, 08036 Barcelona, Spain; 7 Department of Radiotherapy Oncology, Hospital Clínic Barcelona, 08036 Barcelona, Spain; 8 Department of Surgery and Medical Specialities, Faculty of Medicine and Health Sciences, Universitat de Barcelona (UB), 08036 Barcelona, Spain

**Keywords:** biomarker, persistent PSA, liquid biopsy, cell-free DNA, localized prostate cancer

## Abstract

**Background/Objectives:** Currently, the prediction of disease recurrence after radical prostatectomy (RP) in localized prostate cancer (PCa) relies on clinicopathological parameters, which lack accuracy in predicting clinical outcomes. This study focused on evaluating the utility of cfDNA levels and fragmentation patterns as prognostic biomarkers in progressive prostate-specific antigen (PSA) patients, including those with persistent PSA and biochemical recurrence (BR), after primary treatment in localized PCa patients. **Methods:** Twenty-nine high-risk localized PCa patients were enrolled in the study between February 2022 and May 2023. Blood samples were obtained before robotic RP. cfDNA concentration and fragment size were quantified using the Quant-it PicoGreen dsDNA Assay kit and Agilent 2200 TapeStation System, respectively. **Results:** The mean PSA value at diagnosis was 9.4 ng/mL. Seven patients (24.1%) had stage pT2 and 22 (75.9%) pT3. Nine patients (31%) had detectable PSA at the first PSA control six weeks after surgery, and four patients (20%) had BR during a mean follow-up of 18.4 months. No associations were found between cfDNA levels or fragmentation patterns and clinicopathological data. Although not statistically significant, patients with detectable PSA levels post-surgery exhibited higher cfDNA levels and shorter fragments compared with those with undetectable PSA. **Conclusions:** Our study indicated a tendency toward more fragmented cfDNA levels in PCa patients with persistent PSA. Strikingly, biochemical recurrent PCa patients exhibited similar cfDNA levels and fragmentation patterns compared to non-recurrent patients. Further studies exploring liquid biopsy-derived biomarkers in localized PCa patients are needed to elucidate their clinical utility in predicting PSA persistence.

## 1. Introduction

Prostate cancer (PCa) is the second most frequent neoplasm and the fifth most deadly in men, with an estimated incidence of 1.5 million new cases and an estimated mortality of 397,000 in 2022 worldwide [1].

Current clinical practice guidelines for the early detection of PCa recommend a personalized prostate-specific antigen (PSA)-based management for clinical decision-making to improve the risk–benefit ratio of a screening strategy [2]. PCa endorses the stratification of PSA risk according to individual total PSA values and age, with the final time to offer biopsy to patients at increased risk of high-grade disease [3]. PCa is usually diagnosed in the early stages of the disease when the cancer is still localized or locally advanced. Usually, the treatment of localized PCa patients includes a local approach, such as surgery or radiotherapy [4]. Based on PSA values, pathological parameters, and rectal examination, localized PCa patients are classified into low, intermediate, high, and very high risk of progression [5]. High-risk and very-high-risk PCa patients represent around 30% of localized PCa patients, and they have an increased risk of biochemical recurrence (BR) and metastatic progression after initial treatment [6]. Moreover, around 20% of high-risk PCa patients present persistent PSA, defined as PSA > 0.1 ng/mL, within 4 to 8 weeks of surgery [7,8]. Persistent or detectable PSA after surgery is one of the worst prognostic factors associated with oncological outcomes, and it may derive from positive margins, residual benign prostate surgical margins, and especially pre-existing micrometastases [5,9,10]. Current risk classification and recent molecular imaging techniques, such as PSMA-PET performance, might help. However, they cannot reliably identify patients who will present persistent PSA after radical prostatectomy (RP). Moreover, few studies have searched for new biomarkers in detectable PSA patients [9,11]. Therefore, more reliable molecular tools are still needed to identify patients presenting persistent PSA to improve disease management.

In this regard, liquid biopsy techniques are gaining considerable importance in studying molecular biomarkers in several cancer types, including PCa. Liquid biopsy analysis provides cancer-specific information from a straightforward and minimally invasive blood extraction, which can be repeatedly obtained from patients [12]. Different components can be isolated, including cell-free DNA (cfDNA) or circulating tumor cells (CTC), among others. cfDNA consists of highly fragmented nucleic acids secreted in the bloodstream from apoptotic cells—both healthy and cancer cells. High cfDNA levels and short cfDNA fragments (shorter than 150 base pairs; bp) have been proposed as biomarkers of poor prognosis in different urologic tumors [13,14,15,16]. Liquid biopsy research in PCa has been primarily focused on metastatic hormone-sensitive PCa (mHSPC) and metastatic castration-resistant PCa (mCRPC) due to the higher tumor burden, and therefore, liquid biopsy-based components are more commonly found. To our knowledge, the role of liquid biopsy in identifying persistent PSA after RP in localized PCa patients has not been studied.

Hence, we explored the values of cfDNA levels and mean fragmentation patterns in progressive PSA patients, including patients showing persistent PSA and BR. Integrating liquid biopsy-based biomarkers with current risk classification parameters could improve risk management and aid therapeutic decision-making.

## 2. Methods

### 2.1. Patients and Samples

A total of 29 high-risk localized PC patients who underwent robotic RP and lymphadenectomy at our center (Hospital Clinic Barcelona, Barcelona, Spain) were prospectively enrolled in the study between February 2022 and May 2023. The clinical and pathological characteristics of these patients are shown in Table 1. All high-risk patients were staged with PSMA PET/CT scans. Imaging techniques demonstrated that none of the enrolled patients had distant extensions at diagnosis. Patient follow-up was based on serial PSA quantification at different times: six weeks after RP, quarterly during the first year, biannually for the next five years, and annually thereafter. The mean total follow-up time was 18.4 months (5.3–23.2). PSA persistence was defined as PSA ≥ 0.1 ng/mL within 4 to 8 weeks of surgery and BR was defined as PSA > 0.4 ng/mL during follow-up [7,17]. The International Society of Urological Pathology (ISUP) grade is now recommended for use PCa grading, replacing the total Gleason score in the updated guidelines [18]. Notably, ISUP grading allows for splitting a Gleason score of 7 into two prognostically different patterns—3 + 4 and 4 + 3 groups, for ISUP grades 2 and 3, respectively; the first one mostly includes well-differentiated cancers, whereas the latter mostly includes a higher percentage of poorly differentiated cancers associated with a worse prognosis [19]. All participants provided written informed consent (HCB/2013/8753) before being included in this study. The study methodology conformed to the standard set by the Declaration of Helsinki, and was approved by the Clinical Research Ethics Committee of the Hospital Clínic Barcelona (HCB/2022/0542).

One 10 mL EDTA tube of peripheral blood was collected from each patient before robotic RP between May 2022 and July 2023. The blood was stored at 4 °C until processing within the following 4 h.

### 2.2. cfDNA Isolation and Quantification

To separate plasma, blood samples were centrifuged at 3500 rpm for 15 min at 4 °C, followed by plasma centrifugation at 16,000× *g* for 10 min at 4 °C to remove any remaining cells. Plasma samples were then stored at −80 °C until cfDNA extraction.

According to the manufacturer’s instructions, cfDNA was extracted from 1.5 to 4 mL of plasma (depending on availability) using the QIAamp Circulating Nucleic Acid Kit (Qiagen, Hilden, Germany). Plasma cfDNA concentration and mean fragment size were quantified using the Quant-it PicoGreen dsDNA Assay kit QIAamp (Thermo Fisher Scientific, Waltham, MA, USA) and Agilent 2200 TapeStation System (Santa Clara, CA, USA).

### 2.3. Statistical Analysis

ROC analysis was used to establish cfDNA concentration and fragmentation cut-off for BR. The Youden index was used to identify the cut-off with the best sensitivity and specificity. Correlations between liquid biopsy and clinicopathological data were performed using the Mann–Whitney U test, Spearman’s rank correlation coefficient, or the χ^2^ test depending on the nature of the variable. Kaplan–Meier curves were generated and compared using log-rank tests. Cox regression analysis was performed on liquid biopsy and clinicopathological variables to examine their influence on BR. Statistical significance was established at a *p*-value of 0.05. All analyses were carried out with the SPSS software package (27.0.1.0) (IMB SPSS Statistics 25).

## 3. Results

Of the 29 patients, 9 (31%) presented detectable PSA at the first PSA control six weeks after surgery (Table 2). Disease recurrence in these patients was confirmed with imaging techniques, including PSMA PET/CT in five patients; four patients presented local recurrence, three of whom were treated with targeted radiotherapy (RT) combined with androgen deprivation therapy (ADT) and one with salvage lymphadenectomy. The remaining patient had distant recurrence and was treated with ADT. Disease recurrence was unconfirmed in four patients; however, they underwent RT and ADT due to other risk factors. As for treatment responses, all patients showed undetectable PSA levels after treatment.

During follow-up, 4 of the 29 patients (13.8%) developed BR (Table 2). All biochemically recurring patients were re-staged by at least one imaging technique, including MRIs (four patients), PET-choline (one patient), and PSMA-PET scans (one patient). Local recurrence was confirmed in two patients. Recurrent patients underwent salvage RT combined with ADT, with three patients showing treatment response. No patient died during the follow-up.

Overall, the mean cfDNA concentration was 9.34 ng/mL of plasma (SD = 4.75) and the mean of the most common fragment found in cfDNA samples was 149.89 bp (SD = 14.92). No significant associations were found between clinicopathological parameters—such as initial PSA level, pathological stage, ISUP score, *n* status, and surgical margins—and liquid biopsy data—such as cfDNA concentration and fragmentation.

**Figure 1:**
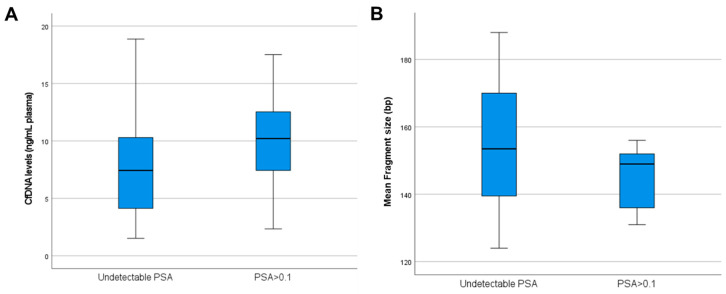
Box plots describing (**A**) cfDNA levels and (**B**) mean fragmentation patterns for PC patients with PSA detectable after surgery.

Patients with persistent PSA tended to have higher cfDNA levels (mean = 10.29 ng/mL of plasma) and shorter fragments (mean = 144.78 bp) than patients with undetectable PSA post-surgery. Despite that tendency, no significant difference was found between both populations (*p* = 0.292 for cfDNA levels; *p* = 0.111 for mean fragment size) (Figure 1). Moreover, associations between liquid biopsy and clinicopathological data did not show any prognostic value in our cohort’s PCa patients with persistent PSA after surgery.

Twenty PCa patients presenting undetectable PSA levels after surgery were followed during a mean of 18.4 months (range: 5.3–23.2) to identify BR. In these patients, mean cfDNA levels and mean fragment size were 8.25 ng/mL of plasma (SD = 4.86) and 155.7 bp (SD = 18.7), respectively. Four patients presented BR during follow-up. cfDNA levels and mean fragmentation patterns based on recurring and non-recurring PCa patients during follow-up can be found in Table 3. Unfortunately, cfDNA levels and fragmentation patterns did not correlate with BR (*p* = 0.9 and *p* = 0.59, respectively).

The median time to BR was nine months (5.3–17.6). cfDNA levels and fragmentation patterns were evaluated through Cox regression analysis to predict RB. The Cox regression model including clinicopathological variables and liquid biopsy data showed no prognostic value of cfDNA levels and mean fragmentation patterns. Kaplan–Meier curves for cfDNA levels and fragmentation patterns found they could not discriminate between recurring and non-recurring localized PCa patients (*p* = 0.9 for cfDNA levels; *p* = 0.456 for fragmentation patterns).

## 4. Discussion

Micrometastatic disease is one of the causes of PSA persistence in a substantial proportion of high-risk PCa patients. However, current risk factors cannot predict patients with micrometastatic disease [5,9]. Liquid biopsy has proven prognostic value for determining clinical outcomes in both urological and non-urological cancer types like bladder, breast, or lung [20,21,22,23]. However, liquid biopsy might be useful for detecting micrometastatic disease after initial treatment and disease persistence in localized cancer settings [10].

To our knowledge, this is the first study to evaluate the utility of liquid biopsy as a biomarker of persistent PSA in high-risk localized PCa patients. We also evaluated the prognostic value of cfDNA for BR. Regarding our results, we did not identify any association between cfDNA levels or mean fragment size and clinical parameters in localized PCa patients. These results agree with those from previous research conducted in this setting. Jung K. et al. did not find significant differences in plasma cfDNA concentration between healthy (*n* = 59) and localized PCa patients (*n* = 62) [24]. Moreover, Chen E. et al. compared cfDNA concentration and fragment size in PCa patients and healthy controls. mCRPC patients (*n* = 122) had significantly higher cfDNA levels than controls (*n* = 31) or localized PCa patients (*n* = 45). Nevertheless, no differences were found between controls and localized PCa patients [25]. Neither study found associations between cfDNA levels, mean fragmentation patterns, and clinical parameters in their cohorts.

Other studies have used different methodologies and liquid biopsy-derived biomarkers, such as circulating tumor DNA (ctDNA) or CTCs, to study PCa patients [26]. Cieślikowski W. et al. focused on CTC count as a predictor of disseminated disease in PCa patients (*n* = 104) [27]. They found that PCa patients who presented with distant metastasis also had a higher CTC count at diagnosis. Pope B. et al. found consistent associations between ctDNA positivity and more aggressive clinical features, like pathological stage or grade, in a localized PCa patient cohort (*n* = 118) [28]. Fei X. et al. found similar tendencies when comparing ctDNA and clinicopathological characteristics [29].

Surprisingly, a tendency was observed when comparing liquid biopsy data between persistent and non-persistent PSA patients. Specifically, the cfDNA mean fragment size tends to be shorter in this subpopulation of patients. It is important to consider that circulating tumor DNA is more fragmented than non-tumor DNA [30]. Patients with detectable PSA post-surgery tend to present micrometastatic disease, which might be reflected in the cfDNA levels found in plasma. To our knowledge, this is the first study to identify that localized PCa patients with detectable PSA post-surgery might have higher cfDNA levels and shorter fragments.

In the context of BR, we found no association between liquid biopsy biomarkers and BR in localized PCa patients, consistently with previous studies. Chen E. et al. found that cfDNA concentration or mean fragment size did not differ across recurring and non-recurring PCa patients [25]. Other liquid biopsy biomarkers used to predict BR in localized PCa patients have been studied. There is confounding data about the utility of CTC in predicting BR, with some authors supporting its association [31] and others refuting it [32,33,34,35]. Moreover, studies showed that ctDNA detection might identify patients with a higher risk of BR, indicating that ctDNA detection could also be used as a biomarker to identify localized PCa patients that might present detectable PSA post-surgery or BR [26,28,29].

Our study acknowledges some limitations. Firstly, our conclusions are based on a tendency found in a relatively small sample size. Secondly, cfDNA level is a non-tumor-specific marker and might be affected by other factors such as tissue damage—like myocardial infarction—or chronic diseases—like diabetes [14,30]. However, this study demonstrated that liquid biopsy-derived components could be useful to identify persistent PSA patients, thus improving disease management in a subpopulation of patients with an unfavorable prognosis.

## 5. Conclusions

Our study showed a tendency towards a higher concentration of and more fragmented cfDNA in persistent PSA localized PCa patients. Moreover, biochemically recurrent PCa patients presented similar cfDNA levels and fragmentation patterns to non-recurring patients. However, further studies exploring liquid biopsy-derived biomarkers in localized PCa patients with detectable PSA post-surgery are needed to elucidate its utility.

## Figures and Tables

**Table 1:** Demographic and clinicopathological data of the studied cohort.

Characteristics	High-Risk Localized PCa Patients (*n* = 29)
Age of diagnosis, median(Q1–Q3)	64 (61–68)
Pathologies of interest, *n* (%)	
No Pathologies	7 (24)
DM	3 (10)
HT	15 (52)
Dyslipidaemia	5 (17)
Initial PSA ng/mL, median (Q1–Q3)	9.4 (5.95–12)
Prostatic volume cc, median (Q1–Q3)	40 (30–54)
ISUP score, *n* (%)	
2	4 (14)
3	10 (34.5)
4	5 (17)
5	10 (34.5)
Pathological Stage, *n* (%)	
pT2	7 (24.1)
pT3	22 (75.9)
pN, *n* (%)	
x	1 (3.4)
0	26 (89.7)
1	2 (6.9)
Affected margins, *n* (%)	17 (58.6)

Abbreviations: PCa, prostate cancer; DM, diabetes mellitus; HT, hypertension; PSA, prostate-specific antigen; ISUP, International Society of Urological Pathology.

**Table 2:** Demographic and clinicopathological data of patients with detectable PSA post-surgery and BR.

Characteristics	Localized PCa Patients with Detectable PSA (*n* = 9)	Localized PCa Patients with BR (*n* = 4)
Age of diagnosis, median (Q1–Q3)	58 (52–65)	64 (60.25–67.5)
Initial PSA ng/mL, median (Q1–Q3)	11 (7.8–26.13)	10.4 (7.3–19.4)
ISUP score, *n* (%)		
2	1 (12)	-
3	4 (44)	2 (50)
4–5	4 (44)	2 (50)
Pathological Stage, *n* (%)		
pT2	2 (22)	-
pT3	7 (78)	4 (100)
pN, *n* (%)		
x	-	1 (25)
0	8 (88)	2 (50)
1	1 (12)	1 (25)
Affected margins, *n* (%)	7 (77.8)	3 (75)
PSA post-surgery ng/mL, median (Q1–Q3)	0.49 (0.26–0.79)	0.05 (0.048–0.058)
Disease recurrence confirmed with imaging techniques, *n* (%)	5 (55)	2 (50)

Abbreviations: PCa, prostate cancer; PSA, prostate-specific antigen; ISUP, International Society of Urological Pathology.

**Table 3:** cfDNA levels and fragmentation patterns in recurring and non-recurring PCa patients.

	Recurring PCa Patients (*n* = 4) Mean (SD)	Non-Recurring PCa Patients (*n* = 16) Mean (SD)
cfDNA levels (ng/mL of plasma)	7.56 (3.2)	8.4 (5.26)
cfDNA fragmentation patterns (bp)	161.25 (17.6)	154.3 (19.2)

Abbreviations: PCa, prostate cancer; cfDNA, cell-free DNA.

## Data Availability

The data presented in this study are available on request from the corresponding author. The data are not publicly available due to hospital policy.
